# Identification of Novel Associations of Candidate Genes with Resistance to Late Blight in *Solanum tuberosum* Group Phureja

**DOI:** 10.3389/fpls.2017.01040

**Published:** 2017-06-15

**Authors:** María F. Álvarez, Myrian Angarita, María C. Delgado, Celsa García, José Jiménez-Gomez, Christiane Gebhardt, Teresa Mosquera

**Affiliations:** ^1^Facultad de Ciencias Agrarias, Universidad Nacional de ColombiaBogotá, Colombia; ^2^Rice Program International Centre for Tropical Agriculture (CIAT)Cali, Colombia; ^3^Department of Genetics and Plant Breeding, Max Planck Institute for Plant Breeding ResearchCologne, Germany

**Keywords:** association mapping, quantitative disease resistance, SNP, late blight, candidate genes

## Abstract

The genetic basis of quantitative disease resistance has been studied in crops for several decades as an alternative to *R* gene mediated resistance. The most important disease in the potato crop is late blight, caused by the oomycete *Phytophthora infestans.* Quantitative disease resistance (QDR), as any other quantitative trait in plants, can be genetically mapped to understand the genetic architecture. Association mapping using DNA-based markers has been implemented in many crops to dissect quantitative traits. We used an association mapping approach with candidate genes to identify the first genes associated with quantitative resistance to late blight in *Solanum tuberosum* Group Phureja. Twenty-nine candidate genes were selected from a set of genes that were differentially expressed during the resistance response to late blight in tetraploid European potato cultivars. The 29 genes were amplified and sequenced in 104 accessions of *S. tuberosum* Group Phureja from Latin America. We identified 238 SNPs in the selected genes and tested them for association with resistance to late blight. The phenotypic data were obtained under field conditions by determining the area under disease progress curve (AUDPC) in two seasons and in two locations. Two genes were associated with QDR to late blight, a potato homolog of thylakoid lumen 15 kDa protein (*StTL15A*) and a stem 28 kDa glycoprotein (*StGP28*).

**Key message**: A first association mapping experiment was conducted in *Solanum tuberosum* Group Phureja germplasm, which identified among 29 candidates two genes associated with quantitative resistance to late blight.

## Introduction

Potato is the most important non-cereal crop consumed worldwide. The main production is centered in Asia with 47.6% of the worldwide production (FAOSTAT, 2015). The most important biotic threat for potato production is the oomycete *P. infestans* causing late blight, a disease that affects potato yield worldwide (Kamoun and Smart, 2005). Usually the pathogen is controlled by frequent applications of pesticides and fungicides that are not environmentally friendly. Research on resistance to late blight has been focused mainly on *R* genes, which confer qualitative resistance. This type of resistance is race-specific, has been overcome in most cases by the pathogen (Bradshaw and Mackay, 1994; Park and Jones, 2009; Yogendra et al., 2014) and is therefore considered not durable for late blight. The alternative to *R* genes is quantitative or polygenic resistance. Many efforts have been undertaken to find quantitative trait loci (QTL) that explain this type of resistance (St. Clair, 2010). In order to be able to take advantage from the QDR via marker assisted selection, it is necessary the identification of genes involved in this type of resistance and their allelic variants (Gebhardt et al., 2007). Since 2002, association mapping was proposed as an option to find genetic variants correlated with traits in a cost-efficient way (Mackay and Powell, 2006).

Diploid potatoes are of economic importance in Andean countries. They are especially cultivated by small farmers. Phureja potatoes also have a high nutritional content (Peña et al., 2015). The diploid *S. tuberosum* Group Phureja had been also used as a source of resistance to late blight (Sliwka et al., 2006, 2010; Tomczyńska et al., 2014; Yogendra et al., 2014). One major resistance gene was found on chromosome IX in Group Phureja germplasm (Sliwka et al., 2006). Quantitative resistance to *P. infestans* segregated in a progeny of a cross between *S. phureja* and *S. stenotomum.* In this progeny, three QTL explained up to 23% of the phenotypic variation, which mapped on chromosomes III, V, and XI (Costanzo et al., 2005). Trognitz et al. (2002) reported a major QTL on chromosome III in a cross *S. phureja* × dihaploid *S. tuberosum* hybrid (Costanzo et al., 2005). These findings confirm that Phureja potatoes are a source of resistance against late blight from both *R* genes and QDR.

The candidate gene approach studies DNA variation in genes known or suspected to play a functional role in a phenotypic trait of interest (Pflieger et al., 2001). Genes identified by differential expression studies are considered as candidate genes (Aghnoum et al., 2009). Known functional candidate genes for disease resistance are genes involved in different steps of the pathogen recognition and signal transduction process. In the pathogen recognition process, one of the most important and broadly studied protein families is characterized by a nucleotide binding and a leucine reach repeat (NB-LRR) domain. *R* genes responsible for the hypersensitive response (HR) against pathogen attack are usually encoded by members of this family (Dodds and Rathjen, 2010). NB-LRR type genes can be found by the NBS-profiling methodology (Linden et al., 2004) or by searching the genome sequence for NB-LRR type genes using bioinformatics tools (Jupe et al., 2012).

Other candidates are genes that are functional in resistance and co-localize with reported resistance QTL, or genes reported to be differentially expressed in resistant versus susceptible plants. QTL mapping for resistance to late blight in various potato genetic backgrounds resulted in approximately 20 QTL distributed on all 12 chromosomes (Gebhardt and Valkonen, 2001; Wang et al., 2009; Danan et al., 2011; Gebhardt, 2013). Within the 20 QTL lay several candidate genes that co-localize. Genes that are differentially expressed in a resistant versus a susceptible interaction might have a direct or indirect role in phenotypic expression of resistance or susceptibility. Interaction of *P. infestans* with potato leaves and tubers has been studied by serial analysis of gene expression (SAGE) or RNAseq (Gyetvai et al., 2012; Draffehn et al., 2013; Gao et al., 2013; Mosquera et al., 2016). Hundreds of genes were found to be differentially expressed. These types of studies are a source for novel candidate genes involved in hypersensitive as well as quantitative resistance to late blight.

Association mapping allows the evaluation of multiple alleles distributed in a germplasm collection for their effects on complex traits that show phenotypic variation in a population. The information of genomes and different technologies of high throughput genotyping opened a gate to association mapping studies in the past 15 years (Abdukarimov and Abdukarimov, 2008). Association mapping is a valid method for working with quantitative traits in plants (Thornsberry et al., 2001; Kraakman et al., 2004; Comadran et al., 2009; Pajerowska-Mukhtar et al., 2009; Simko et al., 2009; Yan et al., 2009; Huang et al., 2010; Neumann et al., 2010; Tian et al., 2011). Association mapping can be performed following two strategies concerning genotyping: (1) selective genotyping at candidate loci and (2) genome-wide association studies (GWAS) (Álvarez et al., 2015). The candidate gene approach for association mapping has been successfully used in potato for several complex traits like resistance to late blight (Pajerowska-Mukhtar et al., 2009), cold sweetening (Fischer et al., 2013), chip color, tuber starch content (Li et al., 2008; Berdugo-Cely et al., 2017), and tuber bruising or enzymatic browning (Urbany et al., 2011). The use of allelic DNA variation at candidate loci for association mapping studies is a strategy to evaluate different alleles in a diverse panel of genotypes. In this research, we used a collection of *S. tuberosum* Group Phureja genotypes in an association mapping study for quantitative resistance to late blight. A set of novel candidate genes was selected based on differential transcript levels in quantitative resistant versus susceptible potato plants (Mosquera et al., 2016). Single nucleotide polymorphisms (SNPs) in the candidate genes were tested for association with late blight resistance.

## Materials and Methods

### Plant Material

We used 104 diploid accessions of the *S. tuberosum* Group Phureja Colombian Central Collection (CCC) that constitutes the Working Collection of the Breeding Program at the National University of Colombia. The genotypes were collected in different potato growing areas of Colombia and were maintained *in vitro* and under field conditions, where they were propagated by tubers.

### Evaluation of Resistance to Late Blight

The 104 genotypes were evaluated for late blight resistance under field conditions, in two growing seasons at two locations in Colombia. The first location was Subachoque in the Cundinamarca department, located at 2,670 meters over sea level (m.o.s.l), the second location was La Union in the Antioquia department at 2,594 m.o.s.l. Crop cycles were carried out during 2010 and 2011. The two seasons and two environments were considered as four environments, divided as follows: (i) Environment one was planted from May to August 2010 at La Union, (ii) Environment two was planted from May to August 2010 in Subachoque, (iii) Environment three was planted from November 2010 to February 2011 in La Union, and (iv) Environment four was planted from December 2010 to April 2011 at Subachoque. The cultivars Capiro and Única were used as susceptible and resistant controls, respectively. The experimental design was random blocks with two plants per block and four repetitions per genotype. The susceptible cultivar Capiro was planted every two rows of evaluated genotypes and in the edges of the plot to increase the inoculum pressure. Weather conditions were registered with meteorological stations. Temperatures ranged from 11 to 16°C and humidity from 81 to 95% at both locations. The infection was natural, as both locations have a high incidence of late blight. The first evaluation was taken 1 month after planting. Disease progression was evaluated weekly during 6–8 weeks, using the percentage of direct visual estimation (PDVE) (Yuen and Forbes, 2009). The area under disease progress curve (AUDPC) was calculated from the PVDE values (Campbell and Madden, 1990). AUDPC values for each location and growing season were calculated, to obtain AUDPC values for four environments (Supplementary Table 1). The virulence spectrum of infecting *P. infestans* was characterized using the reaction of the differentials for eight *R* genes, obtained from the International Potato Center, Lima, Peru^1^. The different isolates were classified as complex because they were virulent for more than three of the eight differentials. The test of virulence was done with the detached leaves test.

### Phenotypic Data Analysis

Statistical analyses were conducted with GenStat 16th, supplied by VNS international. The four field trials were considered as four environments. The rank correlation coefficients matrix between the four environments was calculated and represented graphically, and a principal component analysis (PCA) was performed. Adjusted entry means were calculated using a linear model, considering the four environments as factors in the model:

$$\begin{array}{c} {\text{Y}}_{\text{i}}\;=\;{\text{X}}_{\text{i}}\hat{a}+\;{\text{Z}}_{\text{i}}{\text{b}}_{\text{i}}+{å}_{\text{i}}\\ {\text{b}}_{\text{i}}\sim \text{Nq}(\text{0},\Psi ),{\text{å}}_{\text{i}}\sim \text{Nni}(\text{0},\sigma \text{2}\;\Lambda \text{i}) \end{array}$$

Where: (i) *Y*_i_ is the ni × 1 response vector for observations in the i^th^ group. (ii) *X*_i_ is the ni × p model matrix for the fixed effects for observations in group i, (iii) β is the p × 1 vector of fixed-effect coefficients, (iv) *Z*_i_ is the ni × q model matrix for the random effects for observations in group i. (v) bi is the q × 1 vector of random-effect coefficients for group i. (vi) 𝜀_i_ is the ni × 1 vector of errors for observations in group i. (vii) Ψ is the q × q covariance matrix for the random effects. (viii) σ2Λi is the ni × ni covariance matrix for the errors in group i (Fox, 2002).

### Genotype Molecular Characterization

#### Selection of Candidate Genes

Candidate genes for association analysis were selected from 42,688 differential SNPs, which resulted from a comparative RNAseq experiment between *S. tuberosum* genotype pools with high and low quantitative resistance to late blight (Mosquera et al., 2016). The 42,688 SNPs were distributed in 9,855 genes and showed significantly different allele frequencies between resistant and susceptible genotype pools. Candidate genes were selected using the following criteria in order of relevance: the level of significance for the differential expression between resistance and susceptible pools, using the *p*-value for differential frequency of the SNP allele, the number of differential SNPs per gene and the gene’s position on the Solanaceae function map for pathogen resistance in the GABI (Genome Analysis of the Plant Biological System) Primary Database^2^ (Meyer et al., 2005; Riaño-Pachón et al., 2009).

The genomic positions of the selected genes were compared to QTL for late blight resistance by mapping *in silico* the sequences of markers genetically linked to reported QTL to the potato genome sequence (PGSC v4.03)^3^. Fifty-three genes distributed on all 12 chromosomes were selected, because they contained more than one differential SNP with the Bonferroni corrected *p-*value lower than 0.0001, or/and because they co-localized with a QTL for resistance to late blight (Mosquera et al., 2016). Primers were designed based on the potato genome sequence (PGSC 2011) for the selected genes, considering conserved regions flanking the gene fragment with the highest number of differential SNPs in the comparative transcriptome study (Mosquera et al., 2016) (Supplementary Table 2).

To generate an amplicon from the gene fragment, a PCR reaction was performed in a final volume of 25 μl containing 1X PCR buffer (Fermentas, cat. #B16), 0.2 mM dNTPs (Fermentas, cat. #R0182), 1.5 mM MgCl_2_ (Fermentas, #R0971), 1 unit Tag polymerase (Invitrogen, cat 18038-42), primers 25 mM, 5 ng of DNA. The amplification was done in a thermocycler following the program: 3 min at 94°C, then 30 cycles of 3 min at 94°C, 45 s at the annealing temperature for each primer pair and 1 min at 72°C, final extension at 72°C for 5 min. Amplicons were visualized in 1% agarose gels with ethidium bromide staining prior to sequencing.

#### Collection of SNP Data

Twenty-nine genes distributed on all chromosomes except chromosome IX (**Table 1**) were successfully amplified from genomic DNA of the 104 genotypes. Amplicons were custom sequenced at the Max-Planck-Genome-Center Cologne using the dideoxy chain-termination sequencing method, an ABI PRISM Dye Terminator Cycle Sequencing Ready Reaction Kit and an ABI PRISM 3730 automated DNA Sequencer (Applied Biosystems, Weiterstadt, Germany). Sets of 10 sequences were edited and aligned with DNASTAR software (Burland, 2000), and sequences flanking the SNPs in the 10 genotypes were called. The sequences flanking the selected SNPs were used to call the SNPs with Data Acquisition and Analysis Software DAx (Van Mierlo Software Consultancy, Eindhoven, Netherlands) in the 104 genotypes. Data from DAx software were exported to EXCEL (Excel Office, 2007).

**Table 1:** Genes selected for amplicon sequencing and SNP calling.

Chr.	Start position	End position	Locus ID	Gene annotation
I	558.383	565.761	PGSC0003DMG400019975	Ankyrin repeat-containing protein
I	1.651.485	1.653.969	PGSC0003DMG400032190	Acidic ribosomal protein P1a
I	73.015.925	73.019.833	PGSC0003DMG400000204	Thylakoid membrane phosphoprotein 14 kDa, chloroplastic
I	3.694.969	3.698.957	PGSC0003DMG400016369	Equilibrative nucleoside transporter 1
II	33.873.164	33.877.790	PGSC0003DMG400029694	Eukaryotic translation initiation factor 3 subunit
III	253.765	257.985	PGSC0003DMG400013431	PQ-loop repeat family protein
III	44.499.154	44.501.806	PGSC0003DMG400019959	24 kDa seed maturation protein
III	34.073.643	34.074.711	PGSC0003DMG400016749	TMV-induced protein I
III	61.793.584	61.797.545	PGSC0003DMG400009178	Pectin esterase
IV	2.625.518	2.628.817	PGSC0003DMG400029517	Desacetoxyvindoline 4-hydroxylase
V	5.067.835	5.069.682	PGSC0003DMG400031271	AAA ATPase
V	1.981.074	1.983.124	PGSC0003DMG400000827	Glycosyltransferase, CAZy family GT8
V	2.134.566	2.140.290	PGSC0003DMG400000829	Transmembrane protein TPARL
VI	49.100.842	49.102.333	PGSC0003DMG402016495	Stem 28 kDa glycoprotein
VI	50.409.855	50.412.254	PGSC0003DMG401028933	Ribosomal protein S27
VI	54.345.177	54.347.242	PGSC0003DMG402005942	Endo-alpha-1,4-glucanase
VI	56.859.456	56.860.115	PGSC0003DMG400034939	Thylakoid lumenal 15 kDa protein 1, chloroplastic
VI	50.573.707	50.578.577	PGSC0003DMG401028788	Inducer of CBF expression
VII	53.733.816	53.735.139	PGSC0003DMG400019248	Chlorophyll a-b binding protein 13, chloroplastic
VII	56.364.098	56.365.820	PGSC0003DMG400022241	Photosystem II 10 kDa polypeptide, chloroplastic
VII	54.327.915	54.330.135	PGSC0003DMG400019257	Chloroplast thiazole biosynthetic protein
VIII	46.878.953	46.881.259	PGSC0003DMG400020809	Cytochrome P450 71D11
VIII	5.482.187	5.483.150	PGSC0003DMG400005805	Photosystem I reaction center subunit
X	59.616.753	59.619.226	PGSC0003DMG400007205	Calmodulin
X	56.090.820	56.091.798	PGSC0003DMG400028151	VAMP protein SEC22
XI	41.838.907	41.844.146	PGSC0003DMG400001148	Rubisco subunit binding-protein alpha subunit
XI	43.821.639	43.826.372	PGSC0003DMG400027384	Calmodulin
XI	41.609.684	41.614.152	PGSC0003DMG400008080	CASP^∗^
XII	54.981.145	54.982.086	PGSC0003DMG400016959	ATP synthase delta chain, chloroplastic

*Chromosome (Chr.), start position and end position (bp), locus ID (identification in the potato genome sequence) and functional annotation are shown. ^∗^CASP, are proteins discovered in the CASP (critical assessment of protein structure prediction) project, a world-wide experiment for protein structure prediction.*

### Association Assessment

Association analysis was conducted with Tassel 5 software (Bradbury et al., 2007). Three analyses were done with the general linear model (GLM) and three more with the mixed linear model (MLM). The first GLM included no additional parameters, the second included the population structure (GLMQ), and the third included the PCA population estimation as parameter (GLMPC). The association analysis with the MLM was performed including either kinship alone, kinship and PCA or kinship and population structure. The mixed model used was as follows:

$$\text{Yi}\;=\;\mu \;+\;S_{\alpha }\;+\;{\text{Q}}_{\text{v}}\;+\;\text{e}$$

*Y*i = phenotypic data (adjusted means), *S*α = marker matrix, *Q*v = population structure matrix, *e* = residuals.

Population structure was analyzed with Simple Sequence Repeats (SSR) markers as described by Juyó et al. (2015). Association tests were performed with or without inclusion of population structure linkage groups. The genomic position of each SNP was determined based on the physical chromosome maps according to the PGSC V4.03. The threshold for considering an association significant was fixed at 2.0 –log (*P*). The minor allele frequency (MAF) was tested for two different thresholds values 0.01 and 0.05. With MAF = 0.01 the model could include low frequency alleles in the association test.

QQ plots for each association test with GLM and MLM were compared for goodness of fit. The best fit in the QQ plot was selected for association.

### Gene Co-location with Potato QTL Physical Positions

The position in the potato genome of an associated gene was compared with QTL reported for the chromosome where the associated gene was located. Sequences of markers flanking the reported QTL on the chromosomes III and VI (Danan et al., 2011) were compared trough the BLAST algorithm against the potato genome sequence. Marker sequences were retrieved from the GABI Primary Database^4^ and compared to the potato genome resource website in the blast-n option, with default parameters, against the Potato Genome Sequence Consortium (PGSC) *S. tuberosum* Group Phureja DM1-3 516R44 pseudomolecules (v4.03) (Xu et al., 2011; Supplementary Table 3).

## Results

### Resistance Evaluation

**FIGURE 1:**
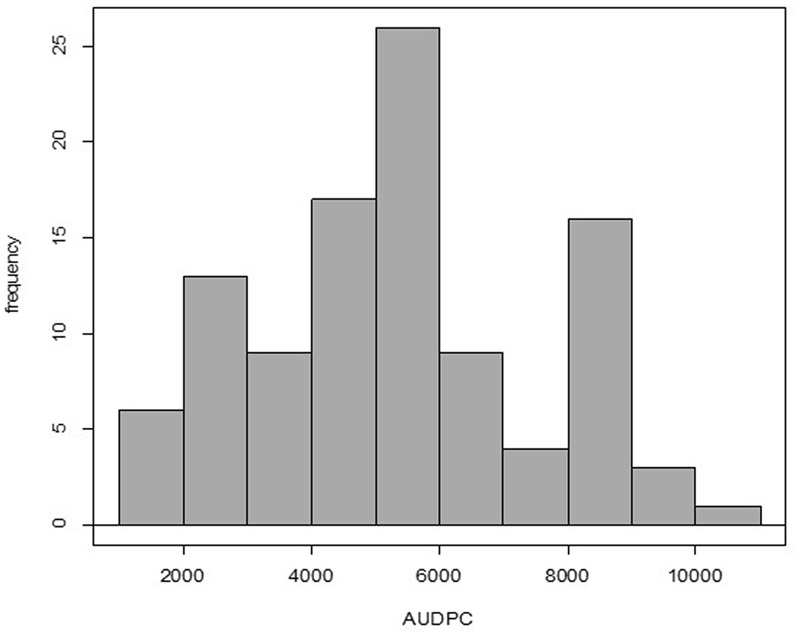
Evaluation of quantitative resistance to late blight in four environments and 104 accessions of *S. tuberosum* Group Phureja. The histogram is based on the adjusted means for area under disease progress curve (AUDPC) values. Shapiro–Wilk normality test, *p*-value = 0.003263.

**FIGURE 2:**
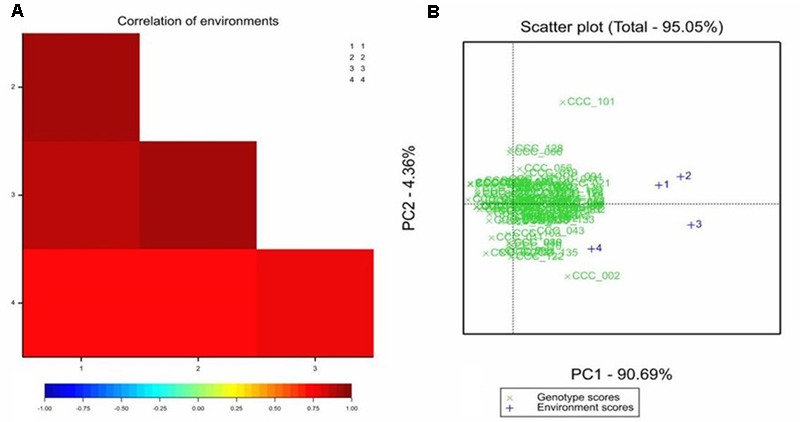
Correlation between areas under disease progress curve (AUDPC) for late blight disease in *S. tuberosum* Group Phureja accessions from the CCC evaluated in four environments. **(A)** Heat map of the correlations between AUDPC values for the environments 1, 2, 3, and 4. The higher the correlation coefficient, the darker the red color. **(B)** The PCA (principal component analysis) for accessions and environment scores, in green, AUDPC values for the accessions and in blue the four environments.

All the genotypes under study were affected in different levels by the pathogen as AUDPC values demonstrate (Supplementary Table 2); no HRs were detected in any genotype. The adjusted means of the AUDPC, calculated from the four environments with the MLM were not normally distributed (**Figure 1**), whereas the residuals of the model were normally distributed (Supplementary Figure 1). The PCA as well as the correlation between AUDPC values from the four environments (Supplementary Table 2) showed that the phenotypic data in all four environments were correlated. The first two principal components of the PCA explained ∼95% of the variation, the third explained 3.06% and fourth 1.364% (**Figure 2**). The first four principal components were sufficient to explain 100% of the variation. Correlation in the PCA graph is determined by the position of each number (environment 1 to 4): the closer they are to each other, the better they are correlated. The grouping of environments 1 to 4 in the PCA plot showed high correlation among environments except for environment 4, which was the least correlated. The resistance or susceptibility levels of the genotypes were similar in all environments, as reflected in the high level of clustering of the genotypes (**Figure 2**). The variation observed in some genotypes that are not grouped such as CCC101 or CCC002 was due to environmental conditions. The genotype × environment interaction was evident by the variation over the different environments of the phenotypic values. For association analysis, we used adjusted means across the four environments (**Figure 1**) considering environmental effects as fixed in the linear model.

#### Candidate Gene Selection

From a list of 1,869 transcripts containing 4,462 SNPs with differential allele frequency between two pools of potato (*S. tuberosum* Group Tuberosum) genotypes with high and low quantitative resistance to late blight, we selected 53 genes, distributed on the 12 potato chromosomes). PCR primers were designed for the 53 genes, PCR conditions were optimized and amplicon quality was evaluated on agarose gels (Supplementary Table 3). Finally, amplicons of 29 genes (**Table 1**) were sequenced and 238 SNPs were called in these genes in the 104 accessions. The putative functions of the selected genes were involved in different cellular processes such as transport, photosynthesis, and protein biosynthesis. The largest group of candidates was related to photosynthesis, especially to photosystems. The 238 SNPs were distributed in 11 of the 12 chromosomes (Supplementary Table 2). Genes on chromosome IX could not be amplified. This chromosome is therefore not represented in the analysis.

#### Association Analysis

**FIGURE 3:**
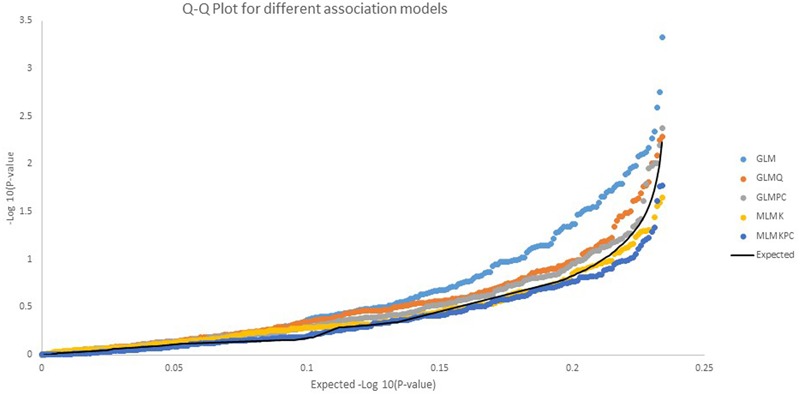
Comparative Q-Q plots for –log_10_ *p*-values for five association models. Three GLM, general linear models: GLM without any other correction parameter, GLMQ (use population structure as parameter *K* = 2), GLMPC (use PCA as a parameter in the model) and two mixed linear models (MLM), MLMK use kinship as a parameter, MLMKPC, use kinship plus PCA The best fit was obtained with the GLM with PCA (GLMPC).

The 238 SNPs were analyzed for association with quantitative resistance to late blight, using the six models described in “Materials and Methods.” The association model with the best fit was the GLM with the principal components (GLMPC) using population structure with principal component as parameter, it was selected by comparing the Q-Q plots of the five of the association models (**Figure 3**). The values for the mixed lineal model with population structure (Q) and kinship (K), MLMKQ, were not possible to fit into **Figure 3**, because its values were out of the range, showing an over estimation of the model due to the interaction of the kinship and population structure.

Two SNPs in two genes were associated with quantitative resistance to late blight (**Table 2**). The SNPs were identified using the name of the locus and their position on the potato pseudomolecules (version 4.03). One SNP of the associated SNP’s was identical with SNPs that had shown differential allele frequencies between resistant and susceptible plants in the RNAseq experiment, based on which candidates were selected.

**Table 2:** Results of association analysis of 238 SNPs in 29 genes for quantitative resistance to late blight in *S. tuberosum* Group Phureja measured as area under disease progress curve.

Chr.	Gene annotation	Primer name	SNP name	SNP identification in the amplicon	SNP position on pseudomolecule (v4.03)	-log_10_ (*P*)	Effect%
VI	Stem 28 kDa glycoprotein	MF12	*StGP28*49101958	ACAT(T/A)TAGT	49.101.958	2.2	11.03
VI	Thylakoid lumenal 15 kDa protein	TM18	*StTL15A56859831*	CCTT(T/C)CCT	56.859.831	2.37	7.0

**FIGURE 4:**
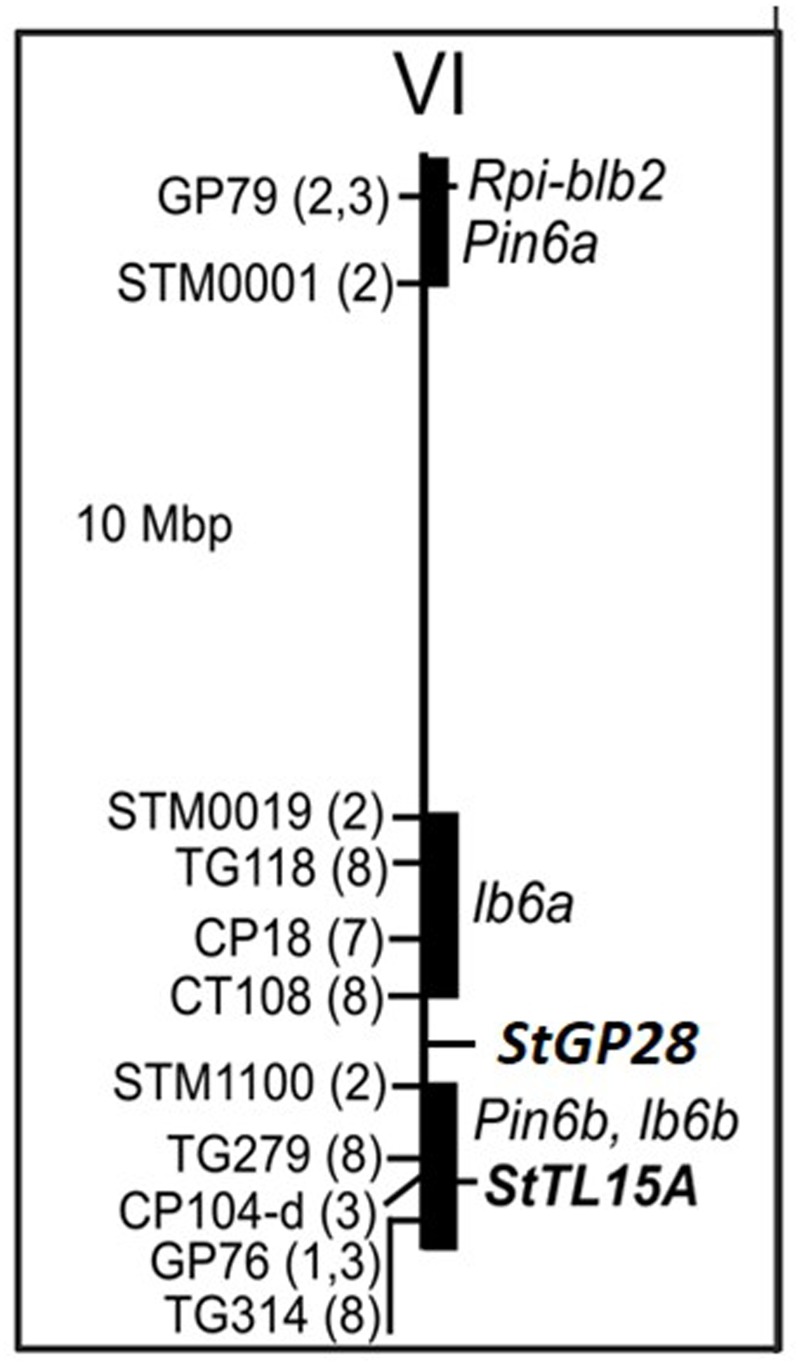
Physical map of potato chromosome VI. Positions of RFLP and SSR markers (see Supplementary Table 3 for details) linked with QTL for resistance to late blight according to the literature are shown on the left. Numbers in parenthesis code for the corresponding references: (1) Leonards-Schippers et al. (1994), (2) Collins et al. (1999), (3) Oberhagemann et al. (1999), (7) Simko et al. (2006), and (8) Brouwer et al. (2004). Black bars highlight the physical segments tagged by QTL linked markers. The approximate positions of potato QRL, *Pin6a and Pin6b* according to the SOL function map for pathogen resistance (http://www.gabipd.org/database/maps.shtml), of tomato QRL *lb6a* and *lb6b* (Brouwer et al., 2004) and the position of the *Rpi-blb2* late blight resistance gene (NCBI accession DQ122125, van der Vossen et al., 2005) are shown on the right along with the positions of the candidate loci *St GP28* and *StTL15A*.

**FIGURE 5:**
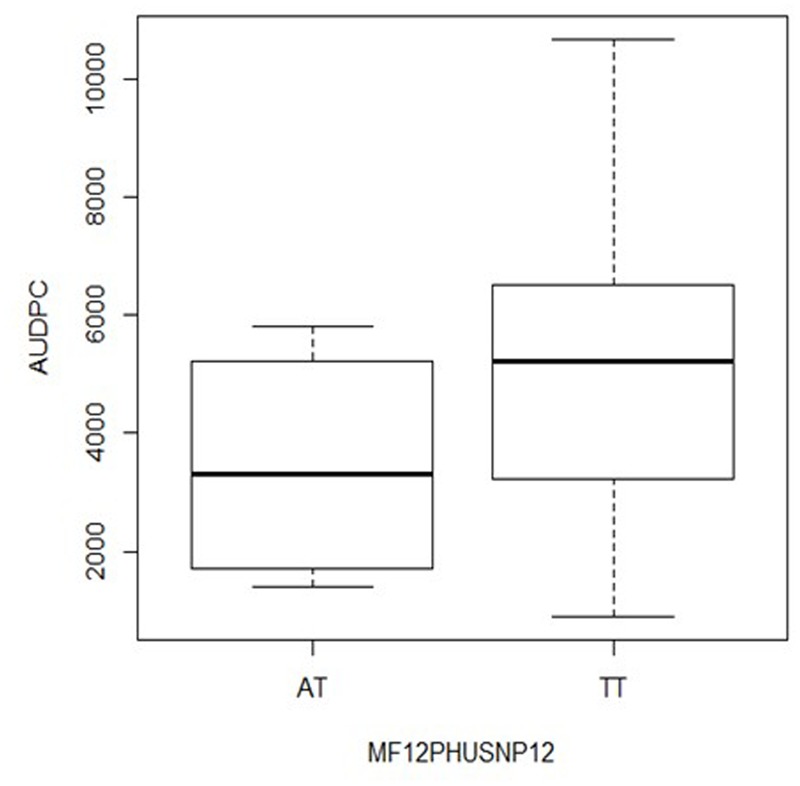
Box plot for the distribution of AUDPC in the genotype classes *AT* and *TT* of SNP *StSGP28G*49101958 (locus PGSC0003DMG402016495 on chromosome VI) in *S. tuberosum* Group Phureja from the Working Collection. The *y*-axis shows AUDPC values and the *x*-axis the two genotype classes. The third genotype class *AA* was absent in the collection.

The first gene was annotated as *Stem 28 kDa glycoprotein* (*StGP28*) on chromosome VI (**Figure 4**) and corresponded to the locus PGSC0003DMG402016495. The associated SNP explained 11% of the phenotypic variation (**Table 2**). The box plot shows that the heterozygous genotype *StGP2849101958 AT* was associated with increased resistance compared with the homozygous genotype *StGP2849101958 TT, which* was associated with susceptibility (**Figure 5**). The SNP lead to a non-conservative amino acid change from tyrosine to phenylalanine in the deduced protein.

**FIGURE 6:**
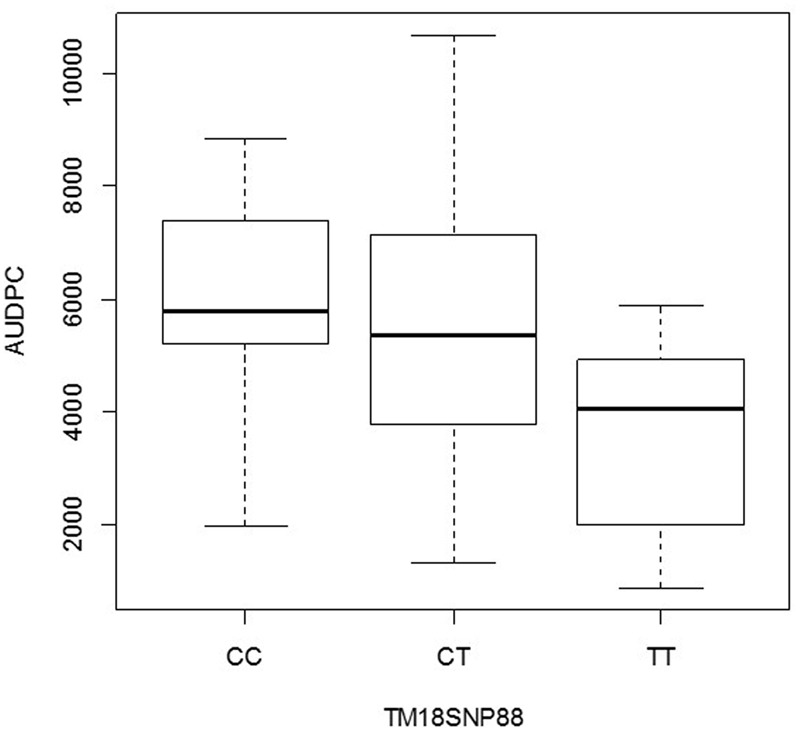
Box plot for the distribution of AUDPC in the genotype classes *CC, TC*, and *TT* of SNP *StTL15A56859831* (locus PGSC0003DMG400034939 on chromosome VI) in *S. tuberosum* Group Phureja from the Working Collection. The *y*-axis shows AUDPC values and the *x*-axis the three genotype classes.

The second gene was annotated as *Thylakoid luminal 15 kDa* protein (*StTL15A*) on chromosome VI (**Figure 4**) and corresponded to the locus PGSC0003DMG400034939. The SNP explained 7% of the phenotypic variation (**Table 2**). The box plot shows that the homozygous genotype *StTL15A56859831 TT* was associated with increased resistance compared to the alternative homozygous genotype *StTL15A56859831 CC* (**Figure 6**). The SNP lead to a non-conservative amino acid change from proline to serine.

The effect of the SNP allele dosage was inferred from the box plots (**Figures 5, 6**). For the gene *StGP28*, the allele dosage effect could not be tested due to the absence of the genotype *AA*. In the gene *StTL15A*, the resistance to late blight increased with the allele dosage (**Figure 6**), showing an additive effect for the marker in *StTL15A*.

Restriction fragment length polymorphism (RFLP) and SSR markers reported to be linked with QTL for late blight resistance on potato and tomato chromosome VI (Leonards-Schippers et al., 1994; Collins et al., 1999; Oberhagemann et al., 1999; Ewing et al., 2000; Bormann et al., 2004; Brouwer et al., 2004; Costanzo et al., 2005; Simko et al., 2006) were mapped *in silico* to the genome sequence (**Figure 5** and Supplementary Table 3). The markers anchored QTL Pin6a to the short arm, QTL lb6a to a 10 Mbp proximal region between 36 and 46 Mbp and QTL Pin6b and lb6b to the 9 Mbp distal regions from 51 to 60 Mbp on the long arm. The *StTL15A* locus was located distal at 56.8 Mbp right within potato QTL Pin6b and tomato QTL lb6b and the *StGP28* locus was located at 49.1 Mbp between the two QTL in the distal arm of chromosome VI.

## Discussion

Here, we report two novel associations between SNPs in two candidate loci and quantitative resistance to late blight in *S. tuberosum* Group Phureja. Both SNP’s are located in expressed genes, one in *StGP28* (Stem 28 kDa glycoprotein) and the other in *StTL15A* (Thylakoid lumenal 15 kDa protein 1). Both genes are located on chromosome VI. The SNPs accounted for 7–11% of the phenotypic variance. Resistance increased or decreased with the allele dosage in *StTL15A*, showing additive allelic effects.

Quantitative resistance is mediated by multiple genes that could influence the level of resistance differentially. It was possible to find additive effects due to the presence of two or more resistance alleles at different loci in a segregating population (Caromel et al., 2005). In our study, additive effects between *StGP28* and *StTL15A* could not be analyzed, since individuals with a combination of the *StGP28* and *StTL15A* resistance alleles were not present in the population. Marker-assisted selection can now be used to obtain genotypes having both alleles and to study their combined effects.

For *StGP28* as well as *StTL15A* the minor frequency haplotype was associated with greater resistance. The most resistant individuals in the population were homozygous for the genotype *StTL15A TT*. The association value for *StTL15A* gene was 237, the highest value found in the association test. This demonstrates that the SNP in *StTL15A* is a promising marker to select for increased resistance in progeny derived from the most resistant genotypes in Working Collection of *S. tuberosum* Group Phureja.

The 29 genes evaluated for association with late blight resistance in a collection of diploid *S. tuberosum* Group Phureja accessions of Colombia were selected based on a comparative RNAseq experiment performed with a set of tetraploid, European *S. tuberosum* Group Tuberosum genotypes (Mosquera et al., 2016). All selected genes contained SNPs with highly differential allele frequencies between groups of plants with contrasting levels of late blight resistance. Even though the list of candidate genes resulted from European tetraploid potatoes it was a suitable tool to discover novel associations with late blight resistance in diploid South American germplasm. Indeed, a different SNP in the *StGP28* gene was associated with late blight resistance in European tetraploid potatoes (Mosquera et al., 2016). This shows that it is possible to use information concerning quantitative resistance of one type of germplasm and environment for another, suggesting that the mechanisms underlying quantitative resistance could be similar in different germplasm and ploidy levels.

Quantitative resistance has been studied in many crops due to the value of quantitative resistance loci (QRL) for breeding applications. Several QRL have been reported in potato on all chromosomes (Gebhardt, 2013). Here, we report two genes associated with quantitative resistance to late blight in chromosome VI.

Three QTL for resistance to late blight have been mapped to the short and long arms of chromosome VI (Leonards-Schippers et al., 1994; Collins et al., 1999; Oberhagemann et al., 1999; Brouwer et al., 2004; Simko et al., 2009). The short arm is a hot spot for qualitative and quantitative resistance to various pathogens in tomato as well as potato (Gebhardt and Valkonen, 2001). It includes *Rpi-blb2*, a functionally characterized *R* gene for resistance to late blight (van der Vossen et al., 2005). The *StGP28* gene maps to position 49.1 Mbp, between two QTL (Ib6a and *Pin6b* – *lb6b)* reported for late blight resistance. The *StTL5A*gene maps to position 56.88 Mbp, within the most distal genome segment from 51 to 60 Mbp on the long arm, which corresponds to potato and tomato late blight QTL *Pin6b* and *lb6b*, respectively (**Figure 4**).

The stem 28 kDa glycoprotein was identified as a vegetative somatic storage protein and is closely related to glycoproteins (Mason et al., 1988). Its specific function in unknown, but it contains domains related to vegetative storage/acid phosphatase.

The thylakoid lumenal 15 kDa protein is one of at least 25 proteins found in the thylakoid lumen compartment of the chloroplast (Kieselbach et al., 1998). Its specific function is unknown. It is a member of the tetratricopeptide repeat (TPR) superfamily, which is highly conserved in cyanobacteria and higher plants. The description of the homologous *Arabidopsis* gene At2g44920 (TAIR^5^) suggests that it functions in carotenoid, chlorophyll, or unsaturated fatty acid biosynthesis, in defense responses, or in response to cold temperature.

*StTL15A* and *StGP28* are expressed in most tissues according to the expression data in the potato genome browser^6^. In a transcript profiling experiment, in which transcript levels of *S. tuberosum* genotype pools with contrasting quantitative resistance to late blight were compared (Draffehn et al., 2013), *StTL15A* (PGSC0003DMG400034939), and *StGP28* were not up- or down-regulated upon infection. However, both genes were found among the 107 transcripts that were differentially expressed in genotype pools with contrasting levels of resistance to late blight. Like several other chloroplasts located proteins, *StTL15A* and *StGP28* were expressed at higher level in genotype pools with higher quantitative resistance compared with susceptible genotype pools, prior to infection with *P. infestans* as well as 1-day post infection (Supplemental Table S10 from Draffehn et al., 2013).

Quantitative resistance is controlled by multiple genes with mostly unknown identity. Here, we report two candidate genes for quantitative resistance to late blight in *S. tuberosum* Group Phureja. To the best of our knowledge this is the first report of association mapping in Group Phureja germplasm. Information generated in previous QTL mapping experiments was valuable to find allelic variations for resistance responses. Mechanisms by which quantitative resistance is controlled might be similar in different potato species, facilitating the transfer of information on genes involved in quantitative resistance between different types of germplasm. The effect of such genes on resistance might vary between different potato germplasm pools, depending on the relative importance of the gene in the resistance response, the distribution and frequency of resistance and susceptibility alleles and genotype × environment interactions.

## Conclusion

Association mapping using candidate genes as markers is a valuable approach to identify genes involved in responses to pathogens under field conditions. Genomic, transcriptomic, and traditional QTL mapping information are useful to find and validate genes associated with late blight resistance. The results generated in this research enable the design of molecular markers that can be evaluated in potato breeding programs.

## Author Contributions

MFÁ designed, planned, and carried out the experiments for genotyping, SNP data analysis, and association mapping. She prepared the draft of the manuscript. MA designed and carried out the phenotypic evaluation in field conditions. CeG addressed the scientific direction of the evaluation to late blight resistance. MD designed and carried out the phenotypic evaluation in field conditions. JJ-G carried out the data analysis of the differential transcriptome experiment and provided the list of candidate genes. ChG provided scientific direction and access to the differential transcriptome analysis data base and contributed to the data analysis. SNP genotyping was performed in her lab at the Max Planck Institute for Plant Breeding Research. She supervised the work and revised the manuscript. TM generated the basic idea for the research in association analysis, planned and designed experiments for genotyping and supervised the project. She designed the manuscript and corrected it.

## Conflict of Interest Statement

The authors declare that the research was conducted in the absence of any commercial or financial relationships that could be construed as a potential conflict of interest.

## Footnotes

1 https://cipotato.org

2 http://www.gabipd.org/database/maps.shtml

3 http://solanaceae.plantbiology.msu.edu

4 http://www.gabipd.org/database/maps.shtml

5 http://www.arabidopsis.org/

6 http://potato.plantbiology.msu.edu/cgi-bin/gbrowse/potato/
