# Supplementary figures and images for: Identification of Novel Associations of Candidate Genes with Resistance to Late Blight in Solanum tuberosum Group Phureja
Source: Front Plant Sci. 2017 Jun 15;8:1040. doi: 10.3389/fpls.2017.01040 (PMC5475386; doi:10.3389/fpls.2017.01040)

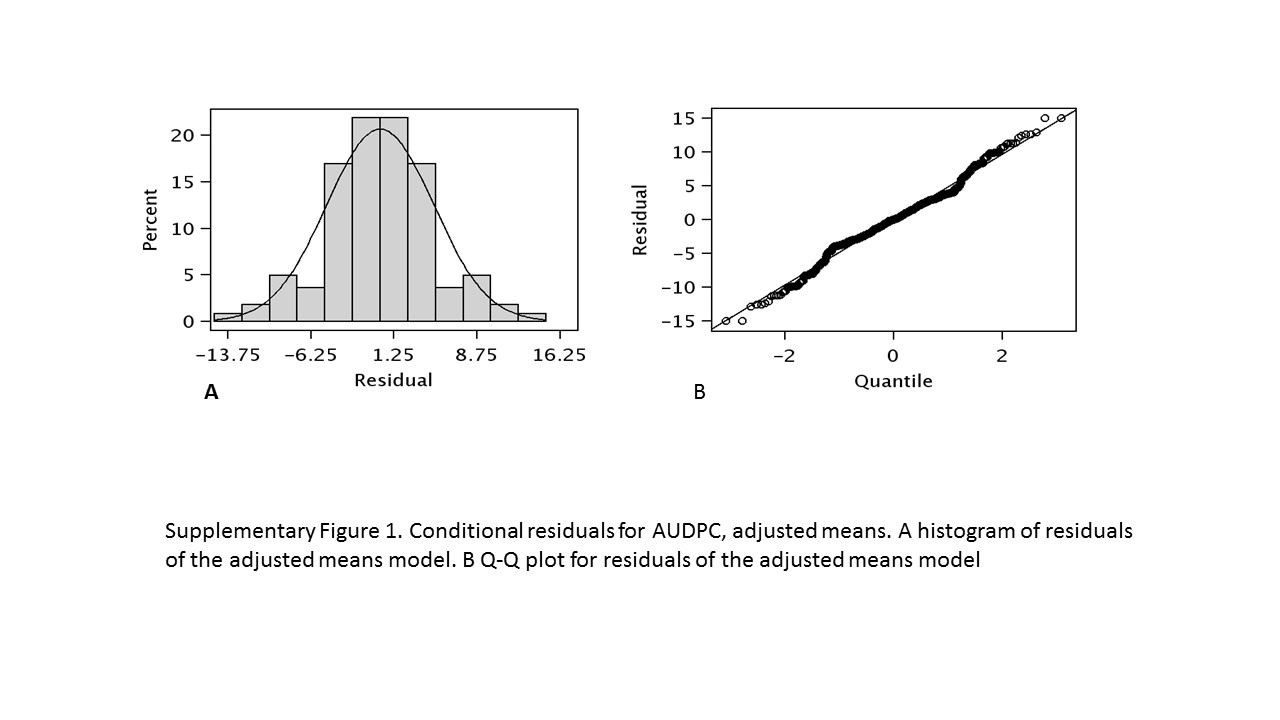

Supplement: Supplementary file 1 [file Image_1.JPEG]
