# Supplementary material for: Identification of Novel Associations of Candidate Genes with Resistance to Late Blight in Solanum tuberosum Group Phureja
Source: Front Plant Sci. 2017 Jun 15;8:1040. doi: 10.3389/fpls.2017.01040 (PMC5475386; doi:10.3389/fpls.2017.01040)
Supplement: Supplementary file 2 [file Table_1.DOCX]

**Supplementary Table 1.** AUDPC values for 161 genotypes of *Solanum tuberosum* group Phureja in four environments.

| Genotype | Environment 1 | Environment 2 | Environment 3 | Environment 4 |
| --- | --- | --- | --- | --- |
| CCC_002 | 9957 | 9556.25 | 13325 | 10228 |
| CCC_003 | 504 | 437.50 | 2045 | 1467 |
| CCC_004 | 1543 | 1256.25 | 3930 | 2089 |
| CCC_005 | 1507 | 2000.00 | 2535 | 1928 |
| CCC_006 | 5571 | 6281.25 | 4995 | 2894 |
| CCC_007 | 1993 | 2037.50 | 3190 | 2906 |
| CCC_008 | 7493 | 9212.50 | 10825 | 3467 |
| CCC_009 | 1943 | 2600.00 | 4730 | 3367 |
| CCC_011 | 1943 | 2587.50 | 5090 | 4222 |
| CCC_013 | 5650 | 6062.50 | 7125 | 4694 |
| CCC_014 | 1593 | 1318.75 | 2390 | 2722 |
| CCC_015 | 5729 | 6350.00 | 10700 | 3167 |
| CCC_016 | 3379 | 3725.00 | 6800 | 3139 |
| CCC_017 | 4921 | 5725.00 | 7475 | 3139 |
| CCC_019 | 6164 | 6975.00 | 8450 | 3194 |
| CCC_020 | 5521 | 6175.00 | 8200 | 3417 |
| CCC_021 | 11579 | 7968.75 | 12950 | 4556 |
| CCC_023 | 3236 | 3906.25 | 6300 | 3806 |
| CCC_024 | 4500 | 3018.75 | 5200 | 6389 |
| CCC_027 | 1893 | 1878.13 | 3460 | 5472 |
| CCC_030 | 2250 | 2193.75 | 2075 | 3306 |
| CCC_031 | 4464 | 4250.00 | 6800 | 3500 |
| CCC_032 | 3071 | 3062.50 | 4550 | 3667 |
| CCC_033 | 5179 | 5812.50 | 7100 | 3472 |
| CCC_034 | 4375 | 4843.75 | 7000 | 3528 |
| CCC_035 | 7250 | 5796.88 | 7700 | 5389 |
| CCC_036 | 5964 | 4937.50 | 7075 | 5361 |
| CCC_037 | 4286 | 4437.50 | 6000 | 3722 |
| CCC_038 | 1893 | 2562.50 | 3200 | 2917 |
| CCC_040 | 4286 | 4000.00 | 6950 | 5500 |
| CCC_041 | 8429 | 10571.88 | 8350 | 6750 |
| CCC_042 | 5679 | 6734.38 | 8275 | 5667 |
| CCC_043 | 6143 | 7500.00 | 8600 | 6917 |
| CCC_044 | 2786 | 2312.50 | 4350 | 2861 |
| CCC_045 | 7857 | 7218.75 | 7800 | 6472 |
| CCC_047 | 5714 | 7031.25 | 8200 | 5611 |
| CCC_051 | 1393 | 1100.00 | 1400 | 1556 |
| CCC_052 | 2357 | 2000.00 | 2650 | 1778 |
| CCC_053 | 2286 | 1537.50 | 3000 | 1472 |
| CCC_056 | 6107 | 6218.75 | 7775 | 2750 |
| CCC_057 | 5714 | 5062.50 | 7150 | 4167 |
| CCC_059 | 4357 | 4500.00 | 4500 | 3361 |
| CCC_061 | 8271 | 8906.25 | 11200 | 6194 |
| CCC_062 | 8321 | 9812.50 | 11300 | 6667 |
| CCC_063 | 8321 | 9843.75 | 11300 | 6278 |
| CCC_065 | 4286 | 4437.50 | 6000 | 3722 |
| CCC_066 | 5714 | 4937.50 | 7100 | 4167 |
| CCC_067 | 2786 | 2312.50 | 4350 | 2833 |
| CCC_069 | 2250 | 2193.75 | 2075 | 3306 |
| CCC_070 | 5179 | 6187.50 | 8200 | 3278 |
| CCC_071 | 5964 | 4937.50 | 7075 | 5111 |
| CCC_072 | 5214 | 6156.25 | 7800 | 3361 |
| CCC_073 | 3429 | 3875.00 | 4100 | 3722 |
| CCC_074 | 1393 | 1100.00 | 1400 | 1556 |
| CCC_076 | 1436 | 2000.00 | 2535 | 2106 |
| CCC_079 | 2786 | 2312.50 | 4350 | 2833 |
| CCC_080 | 6107 | 6218.75 | 7775 | 2750 |
| CCC_083 | 8286 | 8906.25 | 11200 | 5944 |
| CCC_086 | 4143 | 4000.00 | 6900 | 5472 |
| CCC_087 | 8357 | 9937.50 | 11300 | 6278 |
| CCC_088 | 2286 | 1537.50 | 3000 | 1417 |
| CCC_089 | 8321 | 9812.50 | 11300 | 6278 |
| CCC_091 | 8321 | 9781.25 | 11300 | 6333 |
| CCC_094 | 8321 | 9812.50 | 11300 | 4694 |
| CCC_096 | 7857 | 7187.50 | 7800 | 6444 |
| CCC_098 | 3179 | 2156.25 | 7125 | 4333 |
| CCC_099 | 4357 | 4500.00 | 4700 | 3333 |
| CCC_100 | 1393 | 1100.00 | 1400 | 1611 |
| CCC_101 | 11964 | 12875.00 | 9600 | 4361 |
| CCC_102 | 8321 | 9781.25 | 11300 | 5556 |
| CCC_103 | 4143 | 4000.00 | 6950 | 4889 |
| CCC_104 | 5714 | 5093.75 | 7150 | 4194 |
| CCC_106 | 5679 | 6734.38 | 8275 | 5528 |
| CCC_108 | 5286 | 5093.75 | 6325 | 3889 |
| CCC_109 | 1411 | 1178.13 | 1475 | 1611 |
| CCC_110 | 4286 | 4000.00 | 6950 | 5722 |
| CCC_112 | 4214 | 4437.50 | 6150 | 3750 |
| CCC_113 | 5964 | 4968.75 | 7075 | 5417 |
| CCC_114 | 6286 | 4968.75 | 7125 | 5417 |
| CCC_115 | 2179 | 4562.50 | 6150 | 4056 |
| CCC_116 | 5964 | 4906.25 | 7025 | 5194 |
| CCC_117 | 4714 | 4906.25 | 5000 | 3333 |
| CCC_118 | 8321 | 9812.50 | 11300 | 5833 |
| CCC_119 | 2786 | 2312.50 | 4350 | 3333 |
| CCC_120 | 5679 | 6718.75 | 8250 | 5528 |
| CCC_121 | 8429 | 9812.50 | 11300 | 5778 |
| CCC_122 | 4357 | 4078.13 | 6950 | 6417 |
| CCC_123 | 7250 | 5828.13 | 6450 | 5111 |
| CCC_124 | 5614 | 6093.75 | 7175 | 4583 |
| CCC_125 | 1704 | 1375.00 | 2430 | 2806 |
| CCC_126 | 8393 | 9718.75 | 11200 | 5722 |
| CCC_127 | 5179 | 5250.00 | 7000 | 3333 |
| CCC_128 | 5000 | 6531.25 | 5800 | 2222 |
| CCC_131 | 1464 | 2000.00 | 2535 | 2264 |
| CCC_132 | 5929 | 4875.00 | 7025 | 4611 |
| CCC_133 | 4679 | 9812.50 | 11300 | 5778 |
| CCC_135 | 4214 | 5062.50 | 11400 | 4639 |
| CCC_136 | 8429 | 9828.13 | 10600 | 5722 |
| CCC_137 | 5964 | 4937.50 | 7075 | 4889 |
| CCC_138 | 4357 | 4500.0 | 4500 | 3333 |
| CCC_140 | 8464 | 9406.25 | 11200 | 6250 |
| CCC_142 | 5750 | 5000.00 | 6525 | 3944 |
| CCC_143 | 5750 | 5000.00 | 6300 | 3944 |
| CCC_144 | 8464 | 9406.25 | 11200 | 6250 |
| CCC_145 | 8393 | 9406.25 | 11200 | 6250 |
| CRE_UNI | 1543 | 1256.25 | 3830 | 1839 |
| CSU_CRI | 8214 | 9687.50 | 10600 | 5444 |
| CSU_R12 | 8357 | 7437.50 | 9800 | 5444 |
| DIFE_06 | 11036 | 11103.13 | 8350 | 6750 |
| GALERAS | 4286 | 4000.00 | 6950 | 5472 |
| GUANEÑA | 4375 | 4843.75 | 7000 | 3528 |
| LM_8004-01 | 5286 | 5850.00 | 5130 | 2989 |
| LM_8004-04 | 7482 | 9143.75 | 10825 | 4061 |
| LM_8004-07 | 5836 | 6393.75 | 10700 | 4986 |
| LM_8004-08 | 3393 | 3750.00 | 6800 | 3083 |
| LM_8004-11 | 1893 | 2018.75 | 3460 | 1611 |
| LM_8004-13 | 3071 | 3062.50 | 4550 | 3694 |
| LM_8004-14 | 7286 | 5796.88 | 7700 | 5236 |
| LM_8004-17 | 6000 | 5000.00 | 7075 | 4806 |
| LM_8004-18 | 4286 | 4156.25 | 6950 | 5444 |
| LM_8004-20 | 4357 | 4500.00 | 4500 | 3361 |
| LM_8004-22 | 9179 | 8937.50 | 11200 | 6333 |
| LM_8004-24 | 8321 | 9234.38 | 9050 | 6083 |
| LM_8004-25 | 8286 | 8906.25 | 11200 | 5944 |
| LM_8004-26 | 8357 | 9031.25 | 11200 | 5056 |
| LM_8004-27 | 8321 | 9812.50 | 11300 | 4611 |
| LM_8004-28 | 4393 | 4500.00 | 4700 | 3333 |
| MP_14951 | 5036 | 5046.88 | 4640 | 3028 |
| MP_170031 | 5714 | 5656.25 | 6925 | 4611 |
| MP_170041 | 6071 | 5718.75 | 7050 | 5528 |
| MP_170071 | 8536 | 10140.63 | 8350 | 6778 |
| MP_170081 | 6236 | 7531.25 | 8900 | 6694 |
| MP_170091 | 7857 | 7218.75 | 7800 | 6917 |
| MP_170111 | 8357 | 9812.50 | 11300 | 6833 |
| MP_214951 | 6250 | 6250.00 | 8550 | 4319 |
| MP_2622011 | 6000 | 7031.25 | 6000 | 5639 |
| MP_262261 | 6250 | 6218.75 | 7800 | 2861 |
| MP_262271 | 10286 | 5218.75 | 11200 | 6944 |
| MP_262281 | 8321 | 9968.75 | 11500 | 5889 |
| MP_262321 | 4179 | 4375.00 | 6250 | 3750 |
| MP_263122B | 9457 | 9618.75 | 13325 | 10228 |
| TM_802-07 | 1482 | 1240.63 | 1575 | 1611 |
| TM_802-08 | 1704 | 1425.00 | 2530 | 2750 |
| TM_802-09 | 2786 | 2312.50 | 4350 | 1611 |
| TM_802-240 | 8321 | 9687.50 | 11200 | 5722 |
| TM_802-25 | 5000 | 5062.50 | 5800 | 4250 |
| TM_802-258 | 5929 | 4875.00 | 7025 | 4611 |
| TM_802-293 | 1464 | 2000.00 | 2535 | 2264 |
| TM_802-2A4 | 1554 | 2000.00 | 2585 | 2292 |
| TM_802-307 | 9179 | 9812.50 | 11950 | 6194 |
| TM_802-312 | 4571 | 5062.50 | 11400 | 4167 |
| TM_802-329 | 5929 | 4875.00 | 7025 | 4861 |
| TM_802-40 | 1429 | 1209.38 | 1475 | 1611 |
| TM_802-41 | 1429 | 1225.00 | 1525 | 1639 |
| TM_802-42 | 3429 | 3875.00 | 4100 | 3722 |
| TM_802-48A3 | 5964 | 4937.50 | 7075 | 5111 |
| TM_802-49 | 5179 | 6187.50 | 8200 | 3306 |
| TM_802-50 | 5714 | 4937.50 | 7100 | 4389 |
| TM_802-77 | 8271 | 8906.25 | 11200 | 6194 |
| TM_802-85 | 2286 | 1537.50 | 3000 | 1472 |
| TM_802-94 | 1393 | 1100.00 | 1400 | 1556 |
| TM_802-99 | 1893 | 2531.25 | 3200 | 2306 |
