# Supplementary material for: Identification of Novel Associations of Candidate Genes with Resistance to Late Blight in Solanum tuberosum Group Phureja
Source: Front Plant Sci. 2017 Jun 15;8:1040. doi: 10.3389/fpls.2017.01040 (PMC5475386; doi:10.3389/fpls.2017.01040)
Supplement: Supplementary file 3 [file Table_2.DOCX]

**Supplementary Table 2**. Information for the 57 candidate genes selected in potato for the analysis. Chromosome, position of the gene, described function, forward and reverse sequences for primers designed, Tm (Melting temperature) and size of the fragment expected are shown.

| Primer Name | Chr. | Position | Gene | Described function | Forward sequence | Reverse sequence | Tm | Size |
| --- | --- | --- | --- | --- | --- | --- | --- | --- |
| MF18 | Chr01 | 91366 | PGSC0003DMG400019975 | Ankyrin repeat-containing protein | tgt ggt agc gat tct caa agg | ttg tct aac tga agt acc aaa g | 60 | 633 |
| MF19 | Chr01 | 1155020 | PGSC0003DMG400032190 | Acidic ribosomal protein P1a | gct act gga ttg gga tga tc | tag ttc att gtc att gca aac c | 60 | 312 |
| MF20 | Chr01 | 1448697 | PGSC0003DMG401032203 | Extensin | tag ctt aat cac ctg aaa tta tta | cag gtt caa cac aac ttc aat t | 58 | 923 |
| MF22 | Chr01 | 51177602 | PGSC0003DMG400011323 | Defective in induced resistance 1 protein | gt cct cag ctt ata aat tca ac | cga cat ctt aat cat ata gat tc | 60 | 529 |
| TM23 | Chr01 | 66985897 | PGSC0003DMG400032101 | Histidine-containing phosphotransfer protein | tcaaggtcagagaacagattgc | ttactggttggcttcttccatt | 60 | 525 |
| TM24 | Chr01 | 66985897 | PGSC0003DMG400032101 | Histidine-containing phosphotransfer protein | tac taa cca att tta cta cat gat | cat tgt ttg tta ttc ttg atc t | 60 | 694 |
| TM10 | Chr01 | 79159666 | PGSC0003DMG400000204 | Thylakoid membrane phosphoprotein 14 kDa, chloroplastic | tgt aaa ctt tca aga ata gac ag | gta ctt ttt ggg gat atc aag a | 60 | 481 |
| MF21 | Chr01 | 3198504 | PGSC0003DMG400016369 | Equilibrative nucleoside transporter 1 | tcc acc ctg aac caa cgc a | gta caa ctt att att atc cac ag | 60 | 466 |
| TM11 | Chr02 | 55131413 | PGSC0003DMG400029694 | Eukaryotic translation initiation factor 3 subunit | cac tca aag aat aag ctg aat t | cgg att ctt ccg tct ccg | 58 | 842 |
| TM43 | Chr03 | 421782 | PGSC0003DMG400013426 | Unknow protein | atc agg aaa ggc agc aac ag | cga taa ctt cgc ttt caa gtc | 60 | 301 |
| MF1 | chr03 | 470613 | PGSC0003DMG400013431 | PQ-loop repeat family protein | aag agc aca agg cca ggc at | tat tgc tag tat cac gag aat a | 58 | 449 |
| TM13 | chr03 | 962381 | PGSC0003DMG400013460 | Chlorophyll a-b binding protein 3C, chloroplastic | cca cat tac aat aat ctt gta ctg | cat ggc tgc ttc atc cat ggc | 62 | 947 |
| MF2 | chr03 | 4463231 | PGSC0003DMG400012822 | Stem-specific protein TSJT1 | cgt caa ctt ttc aaa gat cat c | ccc ttt act ttc cta atc gac t | 58 | 341 |
| MF3 | Chr03 | 5061391 | PGSC0003DMG400005081 | Nuclear acid binding protein | aag tac caa gtt gga ctc atg | agt tac tga tga taa tag ctt g | 58 | 307 |
| MF6 | Chr03 | 16573450 | PGSC0003DMG400019962 | F-box domain containing protein | gaa tca gaa gat ttt ata gat aac | agg gtt tca caa aga ggg att | 60 | 360 |
| MF4 | Chr03 | 16579344 | PGSC0003DMG400019959 | 24 kDa seed maturation protein | tca aag ttg tgt ttc cat tat ct | tgc gtg tga tct ctt cta tct | 60 | 423 |
| MF5 | Chr03 | 16949238 | PGSC0003DMG400018522 | Thylakoid lumenal 17.4 kDa protein, chloroplast | tca gta cat aca ggg aca ga | caa gta cgt agt agc tta cat | 58 | 279 |
| TM12 | Chr03 | 20417945 | PGSC0003DMG400010170 | Miraculin | ctc aag gat aca tta agg tag c | gct aat ttc att tct cct tgc a | 60 | 1350 |
| TM12b | Chr03 | 20417945 | PGSC0003DMG400010170 | Miraculin | ctc aag gat aca tta agg tag | gct aat ttc att tct cct tgc | 60 | 1350 |
| TM12c | Chr03 | 20417945 | PGSC0003DMG400010170 | Miraculin | ctc aag gat aca tta agg tag | cag agg ttg gcc agt gat tt | 60 | 562 |
| TM14 | Chr03 | 29900386 | PGSC0003DMG400016749 | TMV - induced protein I | agc act ata agc tca aat taa at | agc tct gta agt caa ctt cta c | 62 | 595 |
| TM15 | Chr03 | 45806417 | PGSC0003DMG400009178 | Pectinesterase | att ttt cca caa ctc tcc ttt a | agt cat gca gcg caa cct tt | 60 | 588 |
| TM20 | Chr04 | 2613851 | PGSC0003DMG400029517 | Desacetoxyvindoline 4-hydroxylase | aacacaccgggatctgtatgt | ccattgtcgacacagacatctt | 60 | 913 |
| TM40 | Chr04 | 9021258 | PGSC0003DMG400015010 | proline-rich protein | aat aga cca cta taa tgg tgc | tac aat aat aaa cat cca tat gg | 60 | 1029 |
| TM16 | Chr04 | 55681584 | PGSC0003DMG400006415 | 14-3-3 protein 7 | gtg tac ttg atg gac aat ggt aa | cca atg aag gca att gct aag | 60 | 925 |
| TM35 | Chr05 | 4810910 | PGSC0003DMG400031271 | AAA ATPase | gat cgt aat gct aaa att tga gt | gct tat cgt ctt cac tac caa | 60 | 684 |
| TM33 | Chr05 | 57053638 | PGSC0003DMG400027176 | Transcription factor | tga tgt ttc ttc cct tga aac t | ctt gga ttt gga cat gga cat | 60 | 944 |
| TM34 | Chr05 | 59902172 | PGSC0003DMG400023400 | DNAj | att gca ata ata tgt ttg taa cat t | aca caa atg act gat atg gaa tt | 60 | 378 |
| MF8 | Chr05 | 1981074 | PGSC0003DMG400000827 | Glycosyltransferase, CAZy family GT8 | aag gca aat aat tga agt agg t | ctc aaa gaa gcc atg tcc att | 60 | 513 |
| MF9 | Chr05 | 2044222 | PGSC0003DMG400000812 | Methionine aminopeptidase | gac atg att tag tga tgt ata g | gag ttg aaa cat cca aag tgc | 60 | 343 |
| MF7 | Chr05 | 2134566 | PGSC0003DMG400000829 | Transmembrane protein TPARL | aag aaa tgc aca gat act gaa g | ctt gct tac ccc tat gtt cc | 60 | 675 |
| MF12 | Chr06 | 42623237 | PGSC0003DMG402016495 | Stem 28 kDa glycoprotein | cag aga caa ttt caa aca agt g | gta ctt cgt tgc tca att taa t | 60 | 536 |
| TM41 | Chr06 | 45374719 | PGSC0003DMG401028933 | Ribosomal protein S27 | cta gaa ttg tca ttt ctc tac c | ctt gtt tat tgg taa agt gtg c | 60 | 483 |
| TM17 | Chr06 | 46546227 | PGSC0003DMG400033084 | Chlorophyll a/b-binding protein (cab-12) | act gta acg acg caa gcc t | ttc ctg tca cat tgt gtt gca | 60 | 878 |
| TM19 | Chr06 | 49190783 | PGSC0003DMG400005890 | 16kDa membrane protein | gat cca tcc aat taa tac aat gg | tta tgt tat cca taa gat gca tg | 60 | 565 |
| MF11 | Chr06 | 49595917 | PGSC0003DMG402005942 | Endo-alpha-1,4-glucanase | ccc ata act aac ttc tgg aaa a | cca cta ttt taa gtg cta tca ag | 60 | 582 |
| TM18 | Chr06 | 52355721 | PGSC0003DMG400034939 | Thylakoid lumenal 15 kDa protein 1, chloroplastic | cag ata gat cag cac cag tta | gat tct cag ctt ctg ctt ctg | 62 | 413 |
| MF10 | Chr06 | 45791812 | PGSC0003DMG401028788 | Inducer of CBF expression | aag aag gtc aag act ttc atc | cac tgt aat aat ggc ttt att ct | 60 | 931 |
| TM21 | Chr07 | 44452808 | PGSC0003DMG400018351 | NADPH:protochlorophyllide oxidoreductase | agg tcc ctc cag ttt aag ag | tat tat taa agg aat tcc aag atc | 60 | 916 |
| MF14 | Chr07 | 51164911 | PGSC0003DMG400019248 | Chlorophyll a-b binding protein 13, chloroplastic | ctt agt gaa aat tgt gag gtc | aat tat atg agt ctg ctt cac tt | 60 | 558 |
| TM25 | Chr07 | 51795245 | PGSC0003DMG400022241 | Photosystem II 10 kDa polypeptide, chloroplastic | cga ctg aac tta gaa gat taa g | aac tat gtc tat ata tgt gta gtt | 60 | 957 |
| MF15 | Chr07 | 52092279 | PGSC0003DMG400022249 | Photosystem I reaction center V | cca cat caa ttt cac ata aat ca | tca aaa atg gca tca gct ctg | 60 | 464 |
| TM22 | Chr07 | 52092757 | PGSC0003DMG400022249 | Photosystem I reaction center V | agc tac tgc att aca aac aaa | ca aac ctc cac aca act ca | 60 | 546 |
| TM46 | Chr07 |  | PGSC0003DMG400019257 | chloroplast thiazole biosynthetic protein | gtg ctc cat caa ttt cag caa | cat tct tga gca atc tgt gag | 60 | 548 |
| TM27 | Chr08 | 34096635 | PGSC0003DMG400020809 | Cytochrome P450 71D11 | ttc ttc tca ctg tca cac c | ggt ctt agg tca aaa ttg gag | 60 | 901 |
| MF13 | Chr08 | 34714260 | PGSC0003DMG400030867 | Acyl-CoA-binding protein | tgt caa gca gtg cag taa gg | tca gtt ggt gtc tca tcc tta | 60 | 708 |
| TM26 | Chr08 | 34714464 | PGSC0003DMG400030867 | Acyl-CoA-binding protein | taaggttttgaaccaagttgtta | tccactattcagttggtgtct | 60 | 742 |
| TM47 | Chr08 |  | PGSC0003DMG400005805 | Photosystem I reaction center subunit | tct ctg agt ttg aag aat tgt ta | atg aac tca agt gta ttg gct t | 60 | 848 |
| TM28 | Chr09 | 41638535 | PGSC0003DMG400024285 | Haem peroxidase, plant/fungal/bacterial | cta ctt tga tag tgt tag cat at | gct aat gct aca gag tct cta | 60 | 1187 |
| MF16 | Chr10 | 36079139 | PGSC0003DMG400010283 | Class Ib chitinase | cta ata ata ctt aat tag tgg aca | cca tcc ata act aat act act g | 60 | 462 |
| TM29 | Chr10 | 43300099 | PGSC0003DMG400007205 | Calmodulin | agc tcc agg aca tga taa atg | aca act tta tca gcc gag gta | 60 | 512 |
| TM30 | Chr10 | 48900330 | PGSC0003DMG400028151 | VAMP protein SEC22 | ac ttc cag tgg caa taa act c | att cat tct cca aga cgc tac | 60 | 541 |
| TM3 | Chr11 | 39014650 | PGSC0003DMG400001149 | Allene oxide synthase 2 | ggt tca aaa caa tgg cat taa c | tgc cga gtt ttt cag ctt cg | 60 | 906 |
| TM37 | Chr11 | 39014650 | PGSC0003DMG400001149 | Allene oxide synthase 2 | tga aat cga tag cga aag cag | gaa caa gca agt att ttg gga t | 60 | 740 |
| TM38 | Chr11 | 39022389 | PGSC0003DMG400001148 | rubisco subunit binding-protein alpha subunit | agg tga caa tta cca agg act | gta atg gta ctt ttg gtt tgt c | 60 | 466 |
| TM39 | Chr11 | 39022389 | PGSC0003DMG400001148 | rubisco subunit binding-protein alpha subunit | aac tag ggg aat taa gtg ttc t | tcc gtt cta aag gac aag aca | 60 | 596 |
| MF17 | Chr11 | 41005290 | PGSC0003DMG400027384 | Calmodulin | gaa att act caa tca aaa ggt tt | gcc ttt tcc cat cta tac ata a | 60 | 789 |
| TM32 | Chr12 | 610009 | PGSC0003DMG400015318 | Metallothionein | ttg aaa cgg acg gaa taa gta a | t gtg gag cta caa gtc tga at | 60 | 528 |
| TM31 | Chr12 | 61613990 | PGSC0003DMG400016959 | ATP synthase delta chain, chloroplastic | caa act ccg atc caa acg ca | gct gtg cag caa tat ctt caa | 60 | 596 |
