# Supplementary material for: Identification of Novel Associations of Candidate Genes with Resistance to Late Blight in Solanum tuberosum Group Phureja
Source: Front Plant Sci. 2017 Jun 15;8:1040. doi: 10.3389/fpls.2017.01040 (PMC5475386; doi:10.3389/fpls.2017.01040)
Supplement: Supplementary file 4 [file Table_3.DOCX]

**Supplementary Table 3**. Meta-QTL markers from Danan (2011), position in the potato genome sequence.

| Chr. | Marker | Position in meta QTL cM | Position in PGS | Present in a meta QTL | Number of Meta -QTL | |
| --- | --- | --- | --- | --- | --- | --- |
| 2 | PBA8-11 | 27.20 | 26789000 | no |  |  |
| 2 | StPAD4-1 | 36.69 | 17175685 | yes | 1 |  |
| 2 | StPAD4-2 | 36.69 | 17173003 | yes | 1 |  |
| 2 | At1A9-a | 42.90 | 34695597 | yes | 1 |  |
| 2 | St1.2.4-h | 44.00 | 17659953 | yes | 1 |  |
| 2 | S3e4 | 44.00 | 18501167 | yes | 1 |  |
| 2 | GP23 | 45.09 | 14355524 | yes | 1 |  |
| 2 | At1A7-a | 50.56 | 21827895 | no |  |  |
| 2 | CP48-a | 55.09 | 25025900 | no |  |  |
| 2 | St1.2.3 | 56.10 | 26199200 | no |  |  |
| 2 | GP508 | 56.61 | 25895339 | no |  |  |
| 2 | StNPR1 | 57.12 | 28447838 | no |  |  |
| 2 | At1A3-a | 59.84 | 30133607 | yes | 2 |  |
| 2 | P10b11 | 59.84 | 29683817 | yes | 2 |  |
| 2 | At2A12-b | 62.56 | 31610538 | yes | 2 |  |
| 2 | At1D5-a | 63.12 | 29764405 | yes | 2 |  |
| 2 | GP26 | 64.02 | 46783791 | yes | 2 |  |
| 2 | P2b8-a | 67.61 | 33351345 | yes | 2 |  |
| 2 | S1h5-b | 69.09 | 33667963 | yes | 2 |  |
| 2 | S2d5 | 69.09 | 34078381 | yes | 2 |  |
| 2 | CP65-b | 70.56 | 33875945 | yes | 2 |  |
| 2 | STI0036 | 71.48 | 31851123 | yes | 2 |  |
| 2 | At1A7-b | 73.87 | 21827895 | yes | 2 |  |
| 2 | P2b8-b | 74.64 | 33351345 | yes | 2 |  |
| 2 | STM5114 | 74.68 | 39350024 | yes | 2 |  |
| 2 | P10f5-a | 75.41 | 39652220 | yes | 2 |  |
| 2 | S1b9-d | 76.18 | 41464061 | yes | 2 |  |
| 2 | P8b7-a | 76.18 | 40508576 | yes | 2 |  |
| 2 | S2g2 | 76.82 | 35670036 | yes | 2 |  |
| 2 | P10f5-b | 77.56 | 39652220 | yes | 2 |  |
| 2 | GP216 | 79.03 | 39397901 | no |  |  |
| 2 | S1f6 | 79.20 | 44907195 | no |  |  |
| 2 | GP504 | 83.01 | 44450119 | no |  |  |
| 2 | GP513 | 83.73 | 43585967 | no |  |  |
| 2 | STI0052 | 83.93 | 39772679 | no |  |  |
| 2 | P9f12 | 95.73 | 47138860 | no |  |  |
| 2 | At2F9-b | 96.73 | 47808768 | no |  |  |
| 2 | P2d11-b | 97.73 | 37631212 | no |  |  |
| 2 | GP172-a | 98.73 | 48234953 | no |  |  |
| 3 | Pt2 | 18.68 | 626670 | no |  |  |
| 3 | At1A3-b | 18.68 | 770828 | no |  |  |
| 3 | S1h5-a | 24.68 | 1801673 | no |  |  |
| 3 | S1d4-c | 27.68 | 2773807 | no |  |  |
| 3 | GP510 | 30.68 | 4255392 | no |  |  |
| 3 | Pal | 31.68 | 5522991 | no |  |  |
| 3 | PAL-f | 31.68 | 5522991 | no |  |  |
| 3 | RbcS-1 | 31.68 | 4730295 | no |  |  |
| 3 | P1f3-c | 32.12 | 44825945 | no |  |  |
| 3 | P3c9-c | 33.36 | 7443804 | no |  |  |
| 3 | St1.2.1-b | 33.80 | 21175543 | no |  |  |
| 3 | P3g6-b | 34.23 | 33495817 | no |  |  |
| 3 | At1A9-b | 34.36 | 16887102 | no |  |  |
| 3 | P8f11-a | 35.12 | 49962568 | yes | 1 |  |
| 3 | CP112-a | 35.84 | 34638778 | yes | 1 |  |
| 3 | At2D6-a | 39.76 | 40323606 | yes | 1 |  |
| 3 | At2A2-e | 39.84 | 43019055 | yes | 1 |  |
| 3 | P3d10-a | 47.58 | 43244774 | yes | 2 |  |
| 3 | GP25 | 49.55 | 45121008 | yes | 2 |  |
| 3 | At2D02 | 55.48 | 50907609 | no |  |  |
| 3 | TG74 | 55.48 | 47453004 | no |  |  |
| 3 | P1e9 | 55.48 | 44035279 | no |  |  |
| 3 | CP6 | 57.47 | 49685447 | no |  |  |
| 3 | GP517 | 62.69 | 52405338 | no |  |  |
| 3 | S2f10 | 64.23 | 52616507 | no |  |  |
| 3 | Pha1-a | 65.00 | 57050084 | no |  |  |
| 3 | P7g11 | 70.92 | 55344894 | no |  |  |
| 3 | P7g3-a | 70.92 | 55645375 | no |  |  |
| 3 | S2g5 | 71.70 | 56671913 | no |  |  |
| 3 | St4cl-a | 74.77 | 57754973 | no |  |  |
| 3 | At2A2-a | 79.77 | 43019055 | no |  |  |
| 3 | P1g11-b | 81.77 | 58610995 | no |  |  |
| 3 | S3a3-a | 81.77 | 58877894 | no |  |  |
| 3 | At2B5-e | 83.77 | 55352777 | no |  |  |
| 3 | P3c10 | 89.77 | 60526522 | no |  |  |
| 3 | S3a8 | 94.77 | 62032868 | no |  |  |
| 6 | GP17(b) | 7.50 | 17871387 | yes | 1 |  |
| 6 | At1B7-a | 12.50 | 57713013 | yes | 1 |  |
| 6 | PSTR-g | 15.01 | 59459450 | yes | 1 |  |
| 6 | P1f3-a | 15.50 | 2794913 | yes | 1 |  |
| 6 | GP317-b | 16.00 | 314291 | yes | 1 |  |
| 6 | GP79 | 16.50 | 27400421 | yes | 1 |  |
| 6 | At2B5-c | 16.50 | 40015928 | yes | 1 |  |
| 6 | Cyt-c red 10kD-a | 18.46 | 2717895 | yes | 1 |  |
| 6 | GP164 | 18.47 | 4701496 | yes | 1 |  |
| 6 | P1a1 | 19.26 | 106391 | yes | 1 |  |
| 6 | GP202 | 20.75 | 34312220 | yes | 1 |  |
| 6 | GP249-a | 21.35 | 33647557 | yes | 1 |  |
| 6 | St3.3.13-c | 22.01 | 6588044 | no |  |  |
| 6 | BA5g19 | 22.02 | 21701352 | no |  |  |
| 6 | BA17f21 | 22.02 | 3603053 | no |  |  |
| 6 | At2C9-b | 22.02 | 17908845 | no |  |  |
| 6 | P8a9-a | 22.02 | 51433878 | no |  |  |
| 6 | WUN1 | 22.54 | 50386192 | no |  |  |
| 6 | S1a8-b | 22.60 | 13908634 | no |  |  |
| 6 | P8d7-b | 22.60 | 57217166 | no |  |  |
| 6 | S1g2-a | 22.60 | 8900529 | no |  |  |
| 6 | P3g6-a | 22.61 | 28621585 | no |  |  |
| 6 | P1f11-e | 23.19 | 27288179 | no |  |  |
| 6 | GP164-2 | 23.50 | 4701496 | no |  |  |
| 6 | GP136-b | 23.83 | 31100113 | no |  |  |
| 6 | GP506 | 23.96 | 50602970 | no |  |  |
| 6 | SSR578-a | 24.89 | 45408308 | no |  |  |
| 6 | GP102 | 25.11 | 34574007 | no |  |  |
| 6 | CD67 | 25.43 | 37024388 | no |  |  |
| 6 | TG118 | 27.02 | 39054891 | no |  |  |
| 6 | TG25 | 27.69 | 40931906 | no |  |  |
| 6 | TG54 | 29.17 | 46383900 | no |  |  |
| 6 | TG240 | 29.92 | 46476210 | no |  |  |
| 6 | TG166 | 29.99 | 39670921 | no |  |  |
| 6 | At2B5-b | 30.53 | 40015928 | no |  |  |
| 6 | SSR128 | 31.69 | 41239727 | no |  |  |
| 6 | TG231 | 32.30 | 19118739 | no |  |  |
| 6 | CP18 | 35.01 | 43260224 | no |  |  |
| 6 | STM1050c | 35.58 | 50386192 | no |  |  |
| 6 | At2F2-b | 38.10 | 4151355 | no |  |  |
| 6 | P10g7-b | 41.93 | 45136087 | no |  |  |
| 6 | GP262 | 42.22 | 46611381 | no |  |  |
| 6 | GP215-b | 43.13 | 50464372 | no |  |  |
| 6 | S2a4-b | 44.26 | 47764885 | yes | 2 |  |
| 6 | P8g9-b | 44.26 | 48021179 | yes | 2 |  |
| 6 | St3.3.1-a | 44.26 | 46932436 | yes | 2 |  |
| 6 | Pha1-b | 51.23 | 51924995 | yes | 2 |  |
| 6 | Cyt-c red 33kD | 52.02 | 49670640 | yes | 2 |  |
| 6 | GP89 | 52.93 | 50394411 | yes | 2 |  |
| 6 | St4cl-b | 52.93 | 47997917 | yes | 2 |  |
| 6 | P1g11-a | 55.10 | 50715767 | yes | 2 |  |
| 6 | S1a4-b | 57.42 | 52680942 | yes | 2 |  |
| 6 | S1b2-c | 57.43 | 52386677 | yes | 2 |  |
| 6 | P3c9-a | 57.43 | 53879483 | yes | 2 |  |
| 6 | GP35-f | 59.75 | 24952909 | yes | 2 |  |
| 6 | GP161 | 59.75 | 37886575 | yes | 2 |  |
| 6 | GP24 | 59.97 | 51476114 | yes | 2 |  |
| 6 | CP50 | 60.79 | 53748843 | yes | 2 |  |
| 6 | GP211 | 62.44 | 102414 | yes | 2 |  |
| 6 | At1A9-c | 63.52 | 56393870 | yes | 2 |  |
| 6 | GP233 | 63.84 | 52304476 | yes | 2 |  |
| 6 | GP299-a | 65.24 | 52152955 | yes | 2 |  |
| 6 | GP285 | 65.24 | 53855364 | yes | 2 |  |
| 6 | CP12* | 55.02 | 53950399 | yes | 2 |  |
| 6 | S1g5-a | 66.01 | 55837600 | yes | 2 |  |
| 6 | CP104-d | 66.64 | 55083982 | yes | 2 |  |
| 6 | S1a2 | 67.26 | 55521294 | yes | 2 |  |
| 6 | S1c3-c | 71.02 | 5284896 | yes | 2 |  |
| 6 | TG115* | 71 | 58913746 | yes | 2 |  |
| 6 | At1A6-a | 73.84 | 56419984 | no |  |  |
| 6 | GP36-b | 79.49 | 58987135 | no |  |  |
| 6 | At1B7-b | 80.49 | 57713013 | no |  |  |

*Markers from PoMaMo data base, position in the pathogen resistance maps.
